# Development and Implementation of Element Deletion Algorithm into an Open-Source Software Based on the Fracture Locus of Materials

**DOI:** 10.3390/ma16010187

**Published:** 2022-12-25

**Authors:** Zaki Alomar, Lorenzo Maccioni, Franco Concli

**Affiliations:** Faculty of Science and Technology, Free University of Bolzano/Bozen, 39100 Bolzano, Italy

**Keywords:** fracture analysis, numerical simulation, ductile materials, Code_Aster, lattice structure

## Abstract

An accurate fracture simulation is often associated with how reliably the material model is represented. Hence, many models dealing with the calibration of ductile damage of materials have already been developed to predict failure initiation. Nevertheless, the challenge remains in obtaining an accurate representation of the fracture growth. Herein, an element deletion algorithm is developed and implemented into finite element open-source software. The deleted elements are replaced by new cells made of a virtual low-stiffness material. To better visualize the failure progression, the final model excludes these virtual cells from the representation. The functionality of the algorithm is tested through a series of two-dimensional simulations on three different geometries with a well-known behavior under uniaxial tension. Moreover, the failure response of a three-dimensional lattice structure is numerically investigated and compared against experimental data. The results of the two-dimensional simulations showed the capability of the algorithm to predict the onset of failure, crack nucleation, and fracture growth. Similarly, the onset and the initial fracture region were accurately captured in the three-dimensional case, with some convergence issues that prevent the visualization of the fracture growth. Overall, the results are encouraging, and the algorithm can be improved to introduce other computational functionalities.

## 1. Introduction

**Figure 1:**
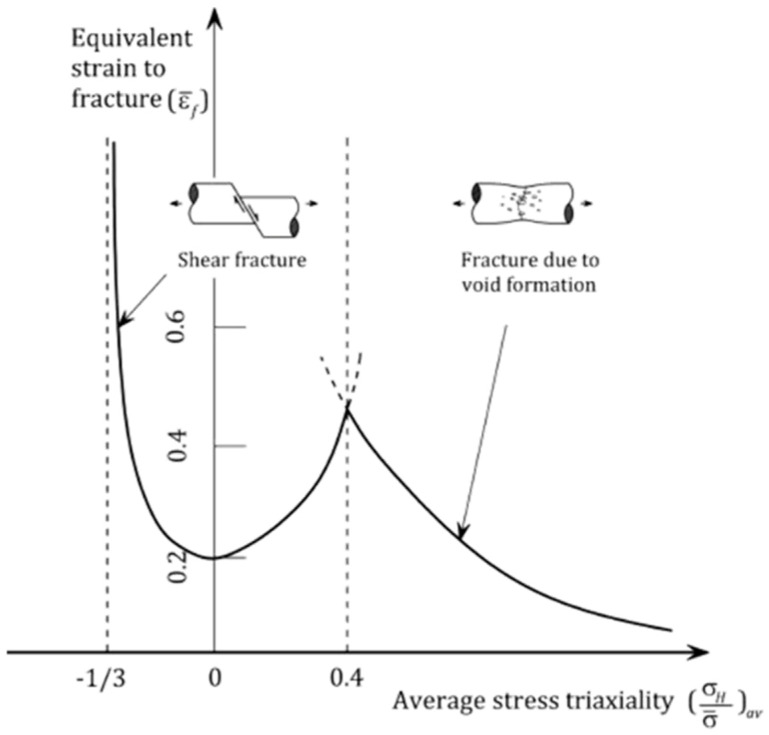
Fracture locus: failure strain vs. average stress triaxiality [5].

The accurate prediction of ductile failure is crucial in many fields, such as the automotive and aerospace industries. Numerical simulations are often employed to predict this failure to save money and time. Particularly, efforts are directed towards the development of accurate ductile damage models that can be calibrated through a series of simple tests and thereafter implemented into Finite Element (FE) codes [1]. The first models proposed by McClintok [2] and Rice and Tracey [3] have shown that the fracture of ductile metals is fundamentally dependent on stress triaxiality. These models have been further improved by Johnson and Cook [4] to include the effects of temperature and strain rate. The conventional Lemaitre’s model takes into consideration the softening effect caused by damage in the stress–strain response. Overall, fracture ductility is the ability of the metal to withstand a large deformation without fracture. Therefore, in all the ductile damage models, the stress triaxiality is defined as a function of the equivalent strain at fracture and it is often not monotonic. The fracture locus presents three different triaxiality regions corresponding to different failure mechanisms, Figure 1. The fracture is governed by shear mode in the region where the stress triaxialities are negative and by void growth for large triaxialities. In between those two regions, the fracture may develop as a combination of the two failure mechanisms: shear and void growth [5].

Bao-Wierzbicki [5] provides a set of equations representing each failure region. $\varepsilon_{f}$ is the equivalent plastic strain at fracture, η is the triaxiality, $\eta_{T}$ is the transition point between the regions, and $D_{\#}$ are the parameters to be calibrated via experimental testing and numerical simulations. That can be easily performed in order to determine the fracture locus of any ductile material. This model, as opposed to other models like Johnson-Cook [4] and Hooputra [6], takes into account failure strain at pure shear conditions. Additionally, the Lode angle is another parameter that has a significant role in the ductile failure of the material, especially at low triaxialities [7]. It is defined as the third invariant of the stress deviator, and it is considered in complex damage model formulation typically to differentiate between the shear stress states in three dimensions [7,8,9,10,11]. Xue [12] and Nahson and Hutchinson [13] proposed models that account for the influence of the third deviatoric stress invariant, and many of the current models follow the same approach in their enhanced model’s formulation.

(1) $$\varepsilon_{f}=\{\begin{array}{cc} \frac{D_{1}}{\left(1+3\eta \right)}+D_{2}, & -\frac{1}{3}<\eta \leq 0\\ D_{3}\eta^{2}+D_{4}\eta +D_{5}, & 0<\eta \leq \eta_{T}\\ D_{6}+D_{7}e^{\left(-D_{8}\eta \right)}, & \eta \geq \eta_{T} \end{array}$$

In the literature, there are many papers dealing with the development and calibration of ductile damage models for all sorts of metals. For instance, Kubik et al. [14] designed a novel compression specimen to test and calibrate the ductile fracture criteria in the negative stress triaxialities. Miscione et al. [15] presented a model dependent on the lode parameter and the triaxiality to explain the origin of chip segmentation and tensile ductile fracture of aluminum alloy. Their model successfully predicted the fractographic features and the chip morphology in metal cutting. Rickhey et al. [16] investigated the influence of the yield criterion and anisotropy on triaxiality. They presented a new method to derive a representative triaxiality value from the average slope of the minor–major principal strain curve. Seo et al. [17] used a simple strain-based model to simulate the ductile crack growth in steel plated with holes and notches. Liano et al. [18] developed a new cyclic fracture model. The model was accurately capable of capturing the global behavior, including the localized damage and the fracture, using the same set of coupled parameters for small and large-scale steel structure components. Nezhad et al. [19] successfully simulated the damage evolution behavior in different ductile sheet metals and shapes utilizing Lemaitre’s ductile damage model under various conditions. Generally, the integration of such models in FE codes remains somewhat limited and not fully exploited. The reason is associated with the high level of expertise needed to properly integrate the damage model along with the element deletion function, particularly. This functionality is almost solely offered by commercial FEA software such as “ABAQUS” by Dassault Systemes [20] and “LS-DYNA” by ANSYS [21], which are considered black boxes for the users. The element deletion function is mainly utilized to remove the highly distorted elements that prevent reaching convergence in an explicit simulation. This method proved to be highly efficient in the simulation of crashes [22], impacts [23,24], machining [25], and damage evolution in materials [26,27]. Aboutalebi et al. [28] utilized a ductile damage model for St12 steel and calibrated its parameters using simple and convenient tensile tests. In their study, the authors applied the element deletion techniques by means of a subroutine available in Abaqus, and they were able to successfully predict the crack initiation, the damage propagation, and the ductile failure. Similarly, Marco et al. [29] used Abaqus to model the fracture of the femur bone and tracked the crack growth by enabling the element deletion technique. The element deletion subroutine was reliable in modeling the crack growth up to a certain extent. Nevertheless, the fracture morphology was not well presented in a long crack path simulation due to convergence difficulties. Baltic et al. [30] used Ansys to demonstrate the potential of ductile fracture locus models in capturing complicated failure modes in pre-cracked structures. They achieved their objectives by adopting a combination of coupled fracture locus theory that relies on fracture strain measurements (averaged experimental strains) and the element deletion approach. Cao [31] used an enhanced Xue coupled damage model combined with element deletion and re-meshing techniques to model crack initiation and growth for various mechanical tests using Forge^®^ FE code. He was able to accurately predict different fracture modes for various load cases. It should be noted that the excessive deletion of elements leads to losses in the overall mass of the system, and in turn underestimation of the expected results.

The integration of the ductile damage model is also possible in a standard implicit simulation. However, the major drawback is the convergence issue which is partially avoided by assigning the element with a low stiffness value instead of completely removing it. In this paper, an element deletion algorithm has been developed following the abovementioned approach. The algorithm was integrated into an open-source Finite Element Analysis (FEA) software, “Code_Aster”. The aim is to predict the fracture nucleation and visualize the damage evolution (crack propagation) with an incremental increase in the load. To achieve this objective, two-dimensional numerical simulations were performed on different specimens made of Ti6Al4V under uniaxial tension. Additionally, a three-dimensional simulation was carried out on a circular-cell-based lattice structure to check the extent of the code functionality. The whole algorithm was written using python scripting language, the same used in the Code_Aster platform.

## 2. Materials and Methods

### 2.1. Framework of the Code

The procedure consists of two main phases. The first phase aims at finding the load or the displacement at which the structure will initially fail. Evidently, that is performed by integrating the appropriate fracture locus of the material being used. Then, in the second phase, the element deletion script is recalled, and the elements whose strain reaches a certain threshold are replaced by a virtual material with low (some order of magnitude) stiffness. Those elements are not visualized in the post-processing in order to better visualize the fracture.

In the first phase, a “for” loop is used to incrementally increase the load/displacement. Typically, the first value is just an initial guess, and then it increases by a small increment in each iteration. The results are continuously being projected to the subsequent simulations using some basics command available in Code_Aster. From the results of each simulation, the triaxiality and the equivalent plastic strain values are extracted at each node. The triaxiality values are used in the equation of the fracture locus (Ti6Al4V) [11] to calculate the theoretical plastic strain. Then, the difference between the actual equivalent plastic strain (from FE) and the theoretical one is calculated and then printed. If the maximum difference is negative, the simulation continues with a larger load/displacement. Conversely, if it is positive or equal to zero, it means that the fracture has been initiated. To save some computational time at the start of this phase, the ideal approach is to significantly increase the load/displacement in each iteration in order to find the range in which the difference of equivalent plastic strain changes from negative to positive. Based on that range, it is then possible to set the optimum starting load and carry on from there.

**Figure 2:**
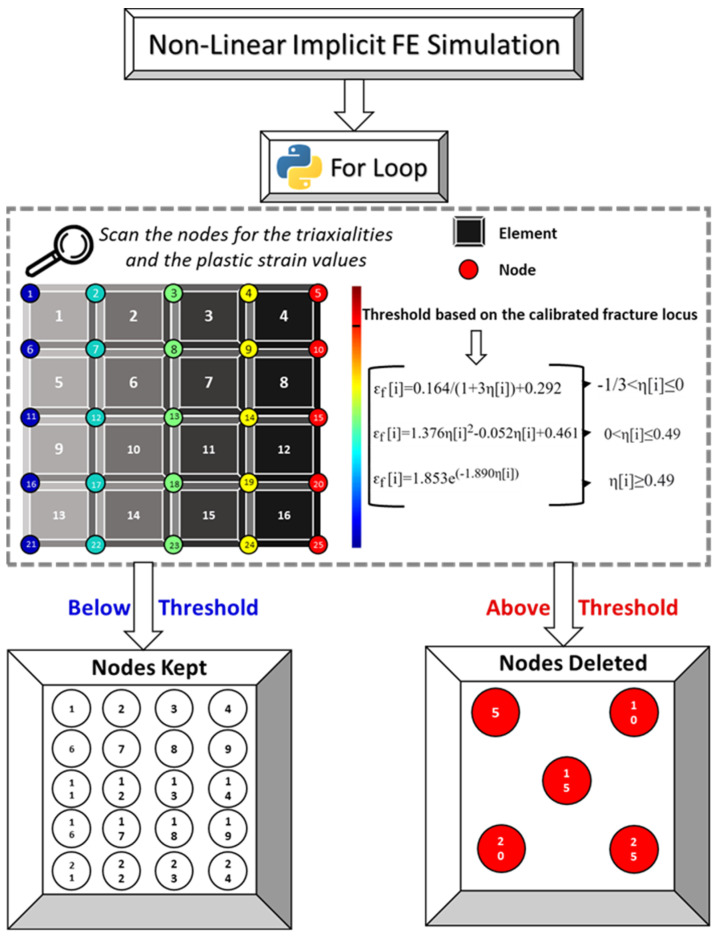
Simplified illustration of the initial part of the code. Dividing the nodes (25 nodes in this example) into two groups (kept and deleted) based on their calculated equivalent plastic strain values compared against the implemented fracture locus of the material used.

**Figure 3:**
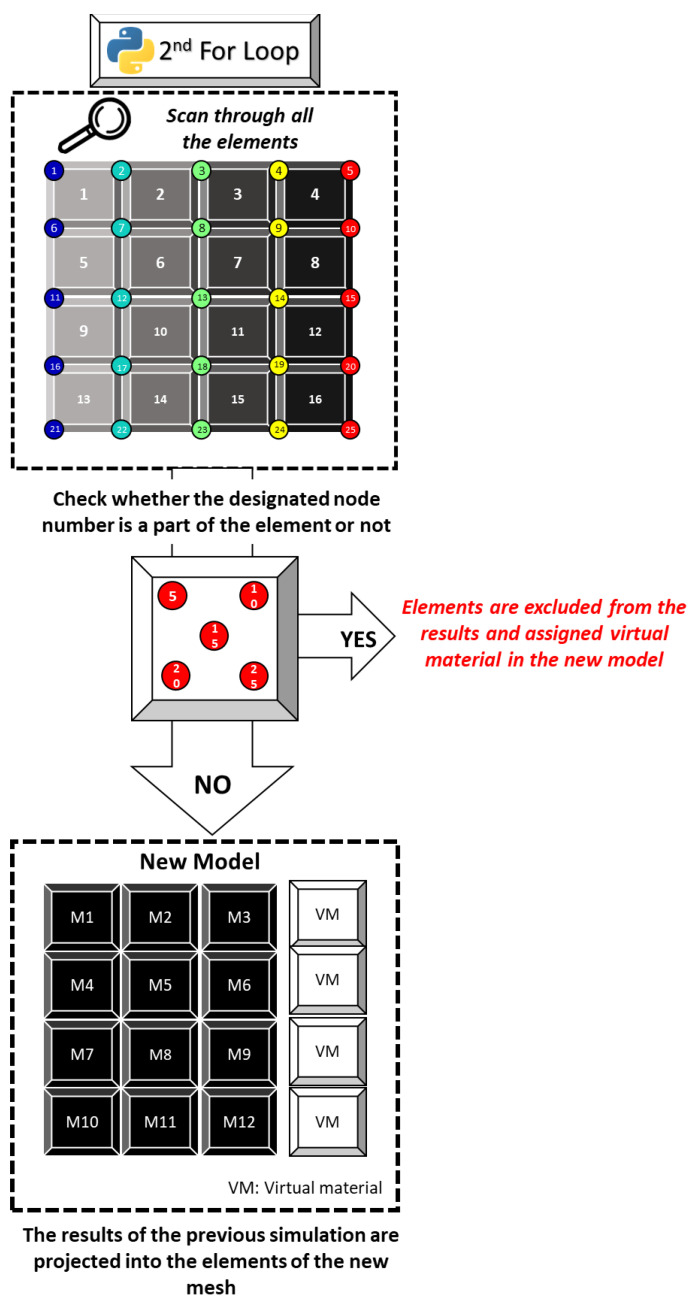
A simplified illustration of the second part of the code. Identifying the elements (out of the 16 elements in this example) corresponding to the nodes’ deleted group (out of the 25 nodes) and then assigning virtual material to these specific elements.

The second phase begins at the initiation of fracture. Since the Code_Aster platform does not have the functionality or the option to remove elements during post-processing, only the elements that are below the threshold are reassigned to a completely new model. In that way, unwanted elements are implicitly removed from the system. Luigi Giaccari [32] successfully used this approach to develop a script that performs structure optimization by material removal. To develop such an algorithm, two functions are created. In the first function, a “for” loop is used to screen through all the values of the difference in the equivalent plastic strain (FE and theoretical) and check whether these values are equal or larger than zero. The designated IDs of the nodes that reach the threshold are then stored in a new list, as shown in Figure 2. To identify the elements that should be kept or deleted, Code_Aster has the option to return the designated number of nodes that constitute each individual element of the mesh. This functionality was essential for the algorithm to actually work. Accordingly, a function was created to scan through all the elements of the mesh and check whether any of the IDs of the nodes obtained are a part of the element connection or not. If the latter holds, the element number is appended to a new empty list as a string with the letter “M” at the beginning (i.e., M1); otherwise, the element number is simply omitted. The new list is then added to the same mesh as a new group which essentially becomes the basis of the new model. Finally, the new group is assigned the actual material, and the rest of the mesh is assigned the virtual material with low stiffness, and only the results corresponding to the new group or model are exported and visualized, as described in Figure 3. The whole framework of the code is also presented in detail through three flowcharts available in the Appendix A.

### 2.2. Numerical Simulation Setup

#### 2.2.1. Two-Dimensional Simulations

**Figure 4:**
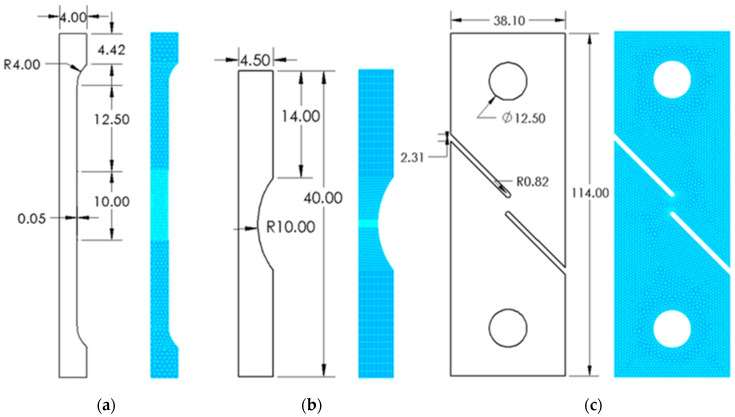
Technical drawing and the mesh of (**a**) round bar, (**b**) round notched bar, and (**c**) pure shear sample. All dimensions are in mm.

To test the effectiveness of the algorithm, implicit static non-linear analysis has been conducted on two-dimensional specimens under uniaxial tension. The implicit static solver is used herein owing to its simplicity and computational efficiency. The simulations are carried out on a simple round bar, round notched bar, and pure shear sample. The design and the dimensions of the specimens are reported in Figure 4. For the round and round notched bar, two-dimensional axis-symmetric modeling is employed along the y-axis (vertical), whereas for the pure shear specimen, a plane strain model is used. Concerning the mesh, quad elements are used to model the round notched bar (max number of segments of 15) and triangular ones to model the round bar and the pure shear samples (max element length of 0.8 mm and 1 mm, respectively). For the round specimens, the mesh is refined in the cross-section where the failure would initiate (max element length of 0.2 mm). The meshes are made quadratic in all the studied cases for better accuracy. It should be noted that a mesh sensitivity analysis has been carried out, and it was shown that with quadratic elements, the results remain relatively unchanged while varying the max element length. The choice of the element length, however, needs to be small for better visualization of the fracture growth as the elements are iteratively deleted while keeping in mind the simulation computation time.

The material properties of Ti6Al4V in its as-built condition are used in these simulations. Particularly, the Poisson ratio is set to 0.3, the modulus of elasticity to 110 GPa, and the yield strength to 992 MPa. The value of the yield strength can be used to check at which load or displacement the material reaches the plasticity region. Moreover, the non-linear behavior of the material was introduced into the simulation using Equation (2) provided by Nalli et al. [33]. The fracture locus limit curve of Ti6Al4V obtained by Giglio et al. [11], presented in Equation (3), has been implemented in the actual ductile fracture algorithm in the framework of Code_Aster. It is of particular importance to assign the right number of nodes that constitute each element of the mesh to run the element deletion algorithm properly. For example, in the case of 2D triangular quadratic mesh, the number is 6, while it is 10 for 2D quadratic quad mesh.

(2) $$\sigma =1300\varepsilon^{0.04}+350\varepsilon \;\left(\mathrm{MPa}\right)$$

(3) $$\varepsilon_{f}=\{\begin{array}{cc} \frac{0.164}{\left(1+3\eta \right)}+0.292, & -\frac{1}{3}<\eta \leq 0\\ 1.376\eta^{2}-0.052\eta +0.461, & 0<\eta \leq 0.49\\ 0.000+1.853e^{\left(-1.890\eta \right)}, & \eta \geq 0.49 \end{array}$$

The mono-axial tension test is performed by applying an incremental displacement at the top edge of the specimens in each simulation. Depending on the convergence, the time step needed is changed accordingly, and it is set in the range of 100 to 1000 steps. The bottom edge is fixed in all directions in translation and rotation. After the execution of the first simulation, the results are projected to the subsequent simulation with the elements to be deleted being replaced by a virtual material (sort of rubber) having a modulus of elasticity of 0.1 GPa. The same process is continued until a complete fracture of the sample is reached, or a convergence problem is reported. The force-displacement values were recorded up to the initiation of the failure and compared to those found in the literature for validation.

#### 2.2.2. Three-Dimensional Simulation

**Figure 5:**
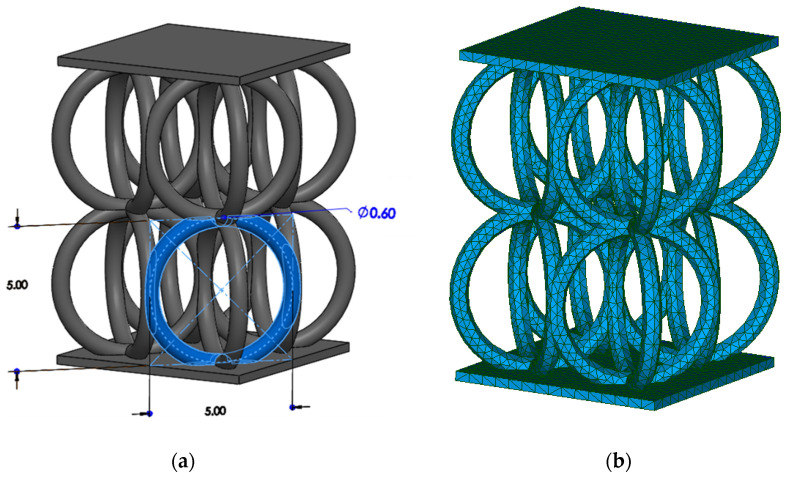
(**a**) A schematic of the CirC model and (**b**) the corresponding 3D tetrahedral mesh.

Metallic lattice structures have gained a lot of traction in various fields due to their superior physical and mechanical properties. Many of the recently published papers studied their failure mechanism and their mechanical response through finite element simulations [34,35,36,37,38,39,40]. Most of the simulations usually focus on the elastic response of the lattice structures or introduce a ductile damage model to investigate their plastic behavior. However, to have a clear representation of the failure behavior of such structures, the implementation of an element deletion approach is almost a must. For instance, struts-based lattices are known to have their struts breaking apart as the load increases, and the latter can only be captured using element deletion techniques. Therefore, the fracture mechanism of a circular cell (CirC)-based lattice structure is herein simulated in order to test the extent of the algorithm’s capability. A 2 × 2 CirC lattice structure is modeled with two plates on the top and bottom where the load and the fixation boundary conditions are applied, respectively. Three-dimensional tetrahedral elements are used to mesh the model with a max element length of 0.5 mm. A schematic of the structure with the corresponding mesh is presented in Figure 5. The same material properties and the fracture locus used in the previous section are employed in the three-dimensional simulation. A uniaxial compression test was simulated by applying an initial displacement at the top plate and then progressively increasing the load.

## 3. Results and Discussions

### 3.1. Two-Dimensional Uniaxial Tension Simulations

**Figure 6:**
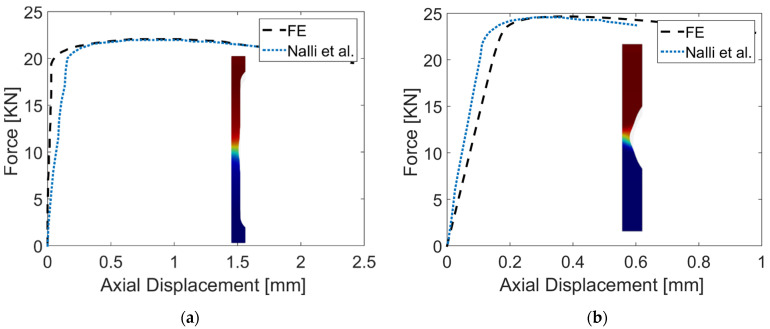
Force displacement curves of (**a**) round bar and (**b**) round notched bar compared with literature data.

A series of 2D simulations were conducted to identify the displacement at which the failure initiates in each sample. The difference between the theoretical (fracture locus) and the FE equivalent plastic strain reaches 0 (onset of failure) at a displacement of 2.4 mm, 1 mm, and 0.9 mm for the round bar, round-notched bar, and the shear sample, respectively. Nalli et al. [33] carried out experimental tests on as-built Ti6Al4V round and round notched bars having the same dimensions adopted here (except the gripers are shorter in this work). The force-displacement curves of both works are compared and presented in Figure 6. There is some discrepancy in the slope of the elastic region. The latter is related to the value of Young’s modulus being used in the simulation. The displacement needed to reach failure is slightly higher for both samples. This can be arguably attributed to the difference between the physical and the virtual model (i.e., surface roughness). Moreover, the calibration of the fracture locus curve itself can be a reason for this increase.

**Figure 7:**
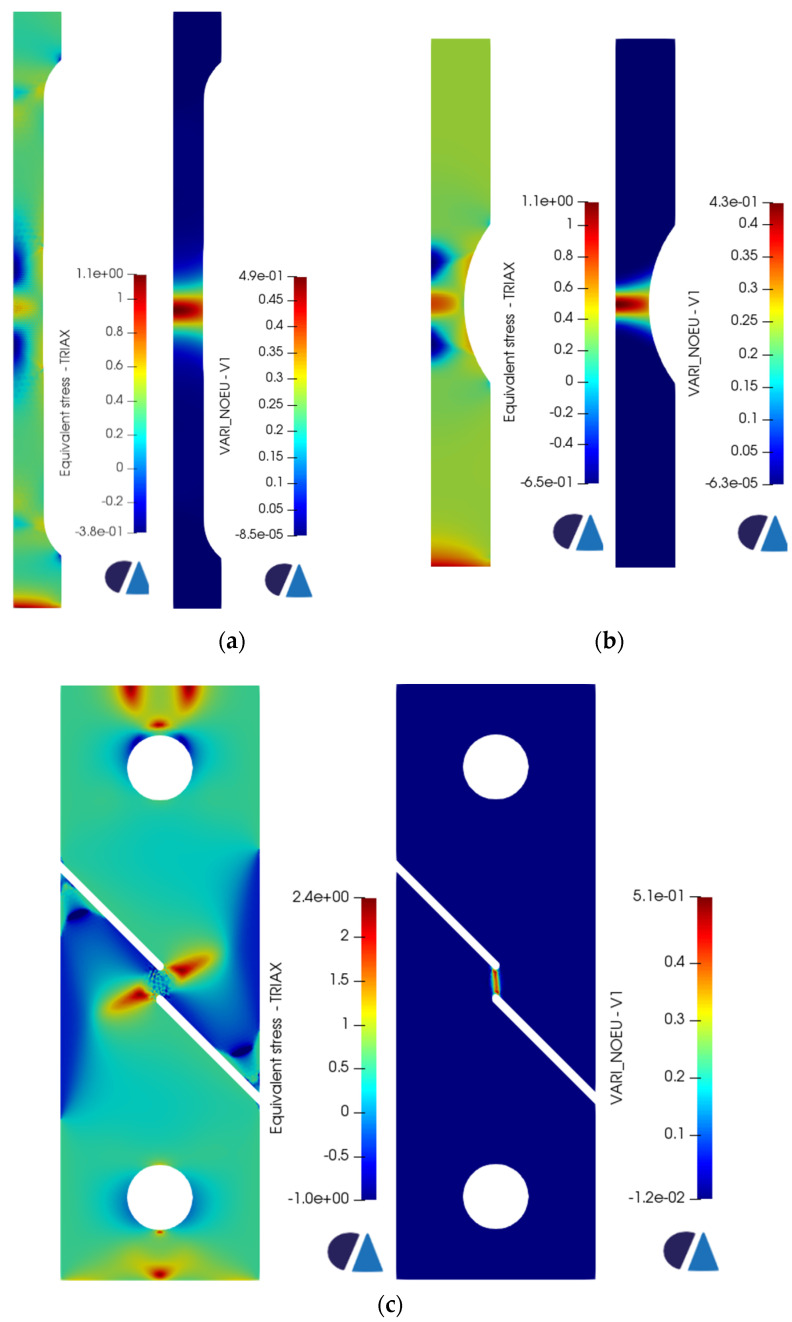
Triaxiality (TRIAX) and Equivalent Plastic Strain (V1) of the (**a**) round bar, (**b**) round notched bar, and (**c**) pure shear sample.

**Figure 8:**
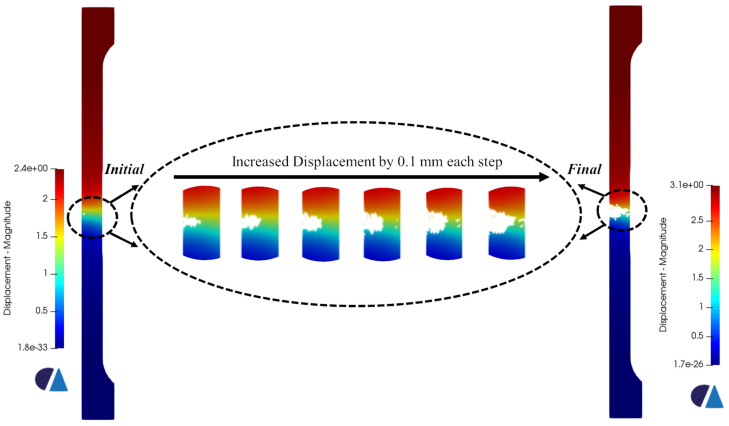
Fracture and failure progression in the round bar with an incremental increase in displacement. The displacement values are in mm.

**Figure 9:**
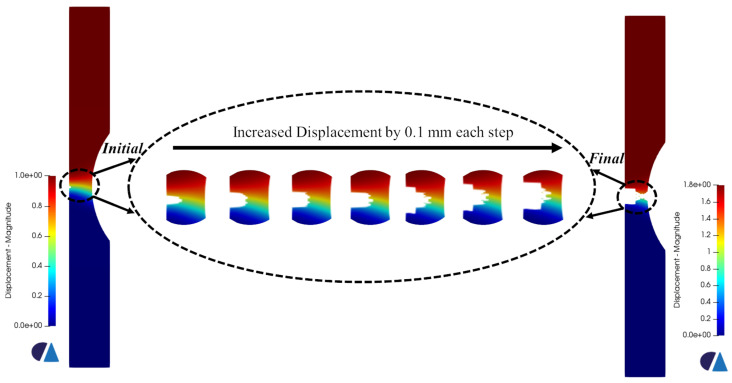
Fracture and failure progression in the round notched bar with an incremental increase in displacement. The displacement values are in mm.

**Figure 10:**
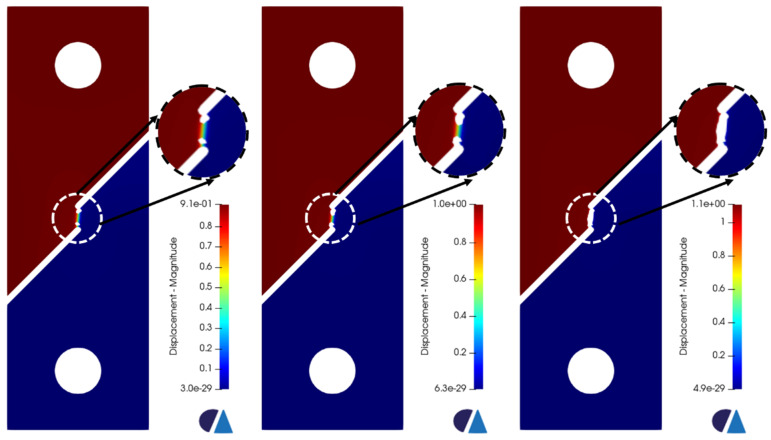
Fracture and failure progression in the shear sample with an incremental increase in displacement. The displacement values are in mm.

Figure 7 presents the triaxiality and the equivalent plastic strain values corresponding to the onset of failure for each sample. Since the Ti6Al4V is a ductile material, void growth is expected under tension in regions of high triaxiality values, specifically larger than 0.49 according to the fracture locus. For the pure shear specimen, the triaxiality values should be around 0. Those values have been clearly observed in the cross-section where the void growth or the fracture will start. The corresponding equivalent plastic strains at the aforementioned regions were obviously the highest as well. Upon reaching the onset of failure, the element deletion algorithm identifies the elements that should be assigned the virtual material’s properties (based on the fracture locus curve). An incremental increase in displacement of 0.1 mm was applied for all the samples in each new simulation. Accordingly, a new group of elements is assigned low-stiffness material properties in each iteration. The iterative process stops once the samples break completely. Figure 8, Figure 9 and Figure 10 show the progression of failure as the displacement increases in each simulation. A total displacement of 0.7, 0.8 and 0.2 mm was needed to reach the complete fracture of the round bar, round notched bar, and the shear sample, respectively. It can be observed from the results that the failure path is well-predicted in the three samples. The fracture does indeed initiates in the middle of the round, and the round notched bar as a void (in the regions of high triaxiality), and that void grows laterally, generating a big crack which in turn leads to the complete rupture of the part. Furthermore, the fracture occurs about the principal plane, which conforms well to the fracture often obtained in physical tensile testing of ductile materials. On the other hand, the shear sample breaks in the regions of the zero-triaxiality as expected. Overall, the element deletion algorithm fulfills its functionality with good accuracy in predicting the initiation location of the failure and its path progress.

### 3.2. Three-Dimensional Uniaxial Compression Simulation

**Figure 11:**
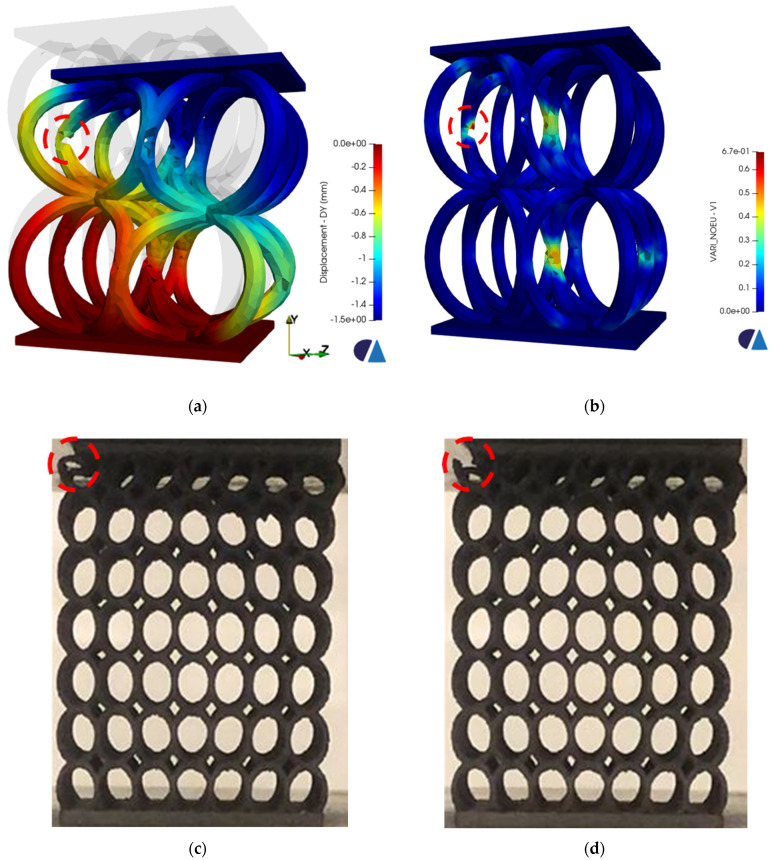
A comparison between the failure outcomes obtained via numerical simulation with the newly implemented algorithm and experimental ones. (**a**,**b**) show the displacement and the corresponding equivalent plastic strain acquired through 3D simulation, as well as the region where the fracture is initiated, which is marked by a red circle. (**c**,**d**) are images taken from previous experiments [41] for the same, but bigger lattice structure with the region of initial and complete fracture also marked by the red circle, respectively.

A couple of three-dimensional simulations were successfully carried out before convergence issues were faced. At a total displacement of 1.5 mm. the fracture of the struts occurred at the middle region of the unit cells, as depicted in Figure 11a,b. Since the fracture mechanism of the CirC-based lattice structure is well known from previous experimental work [41], a comparison with the numerical results was possible to make, noting that the specimens of the experiment were bigger in terms of cell number, yet the dimensions are the same. The CirC lattice structures under compression tend to lean sideways, and the unit cells opposite to that side experience a concentration of stresses that eventually lead to their fracture. Subsequently, a crushing of the whole layer occurs, and the same fracture mechanism is perceived in each layer until densification. Figure 11 shows that numerical simulations coupled with the element deletion code were successfully able to predict the region of the fracture (marked by red circles) when compared with the experimental results. Moreover, a relatively good agreement is obtained between the numerical displacement (1.5 mm) needed to initiate fracture and the experimental one (1.4 mm) for the CirC with 0.6 mm wall thickness. The main issue is the continuation of the simulation and the overcoming of the convergence difficulties. Perhaps the instantaneous fracture of the whole layer afterward generates orphan elements that prevent the simulation from running. Additionally, it has been shown experimentally that when the first layer collapses, it merges with the subsequent layer. This can be quite tedious to model especially using implicit simulations.

Some limitations should be addressed with this algorithm. First of all, mesh quality is crucial to make this code work, and a mesh sensitivity analysis should always be performed. Linear meshes seem to yield incorrect triaxiality values unless the mesh is well-refined. It is suggested to always use quadratic mesh in such simulations. Moreover, hybrid meshes (a combination of triangular and quadrangular elements) are not recommended at this point of the study. For three-dimensional simulations, the algorithm works well for simple cases, but convergence issues may be faced for complex geometries. Moreover, three-dimensional simulations are computationally demanding, and with the element deletion code, this will be more noticeable due to the numerous simulations needed (iterative). Application-wise, it is best suited for predicting the onset of failure, the crack location, and the progression path to a certain extent. Since no energy transfer model is implemented in this approach, the simulation of long crack paths could provide inaccurate results. For the simulation of a long crack path, the extended finite element method (XFEM) is more adequate as it allows to split the finite elements instead of deleting them with little to no re-meshing needed. Lastly, deleting elements completely instead of replacing them with virtual material is rather difficult in implicit simulations. Theoretically, this approach fits better with the explicit type simulations due to the ease of convergence. This would be investigated thoroughly in the future to broaden the functionality of this code.

## 4. Conclusions

In this paper, an element deletion algorithm is developed and implemented into an open-source software, “Code_Aster”, to predict the fracture initiation and the damage evolution as the load increases. The code introduces the fracture locus of a specific ductile material (Ti_6_Al_4_V) and identifies the elements which exceed the fracture locus curve. Those elements are assigned with a virtual material of low stiffness, and they are excluded from the results outputs. The code functionality was tested through a series of two-dimensional implicit numerical simulations on three different specimens: round bar, round notched bar, and pure shear sample under uniaxial tension. Moreover, a three-dimensional simulation of a circular-cell-based lattice structure was carried out, and its failure response was compared against experimental results. The outcomes of this research are encouraging and can be summarized as follows:

- Using the developed algorithm, it was possible to predict well the location and the onset of failure for the three samples. The force-displacement curves and the displacement at failure were compared with experimental data from the literature, and they were in relatively good agreement, yet with some discrepancies associated with numerical errors and surface finish.
- The progression of failure was captured accurately with the incremental increase in displacement. The fracture along the principal plane was observed in the round bars, and the fracture of the shear sample was an accurate representation of a similar physical test.
- The three-dimensional simulation shows a good agreement between the onset of failure and the initial fracture region of the lattice structure. However, the complete failure response was not possible to obtain due to convergence issues.
- There are limitations to this algorithm associated with the type and quality of the mesh. Moreover, it is better to avoid the simulation of long crack paths as there is no energy transfer model embedded.

The functionality of this algorithm can be significantly expanded, and the use of the fracture locus as a criterion for the element deletion is just an example. Other failure criteria can be implemented into the open-source software utilizing the same framework. Additionally, it is possible to use a similar algorithm in other fields of computational modeling, such as topology optimization or thermal analysis, with temperature being the criterion for failure. With the freedom offered by the FE open-source software, the options are limitless, and the presented algorithm can be tailored to fulfill a wide variety of research objectives.

## Figures and Tables

**Figure A1:**
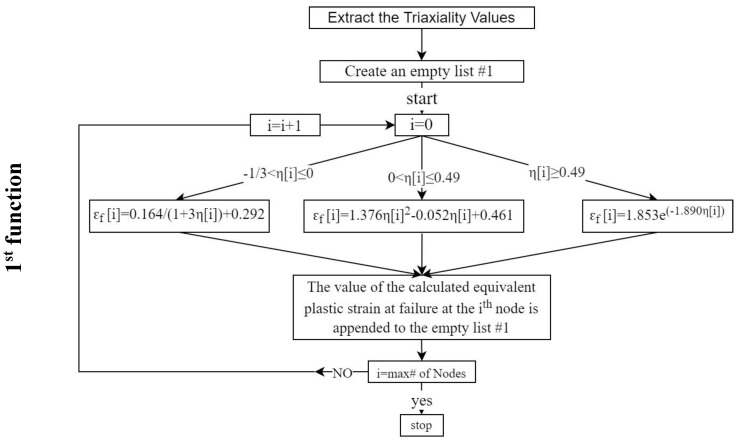
Flow chart describing the method in which the theoretical plastic strain at fracture of each node is calculated.

**Figure A2:**
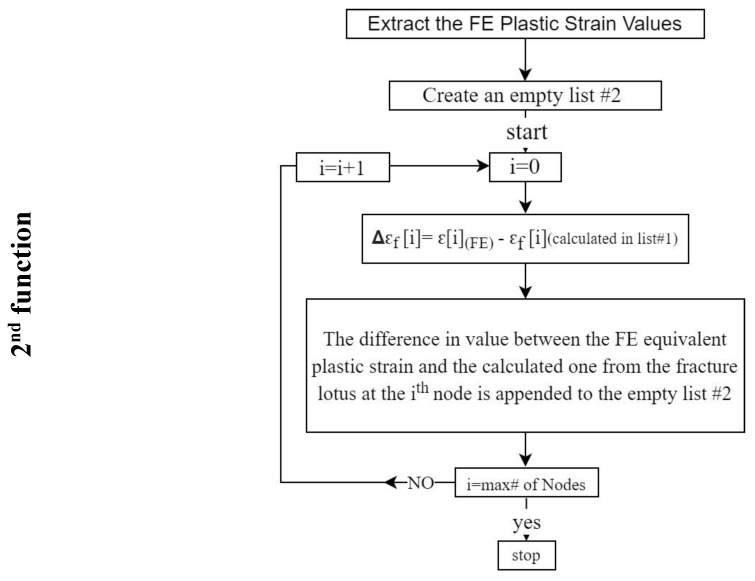
Flow chart describing the method used to identify the difference between the FE and the theoretical equivalent plastic strain.

**Figure A3:**
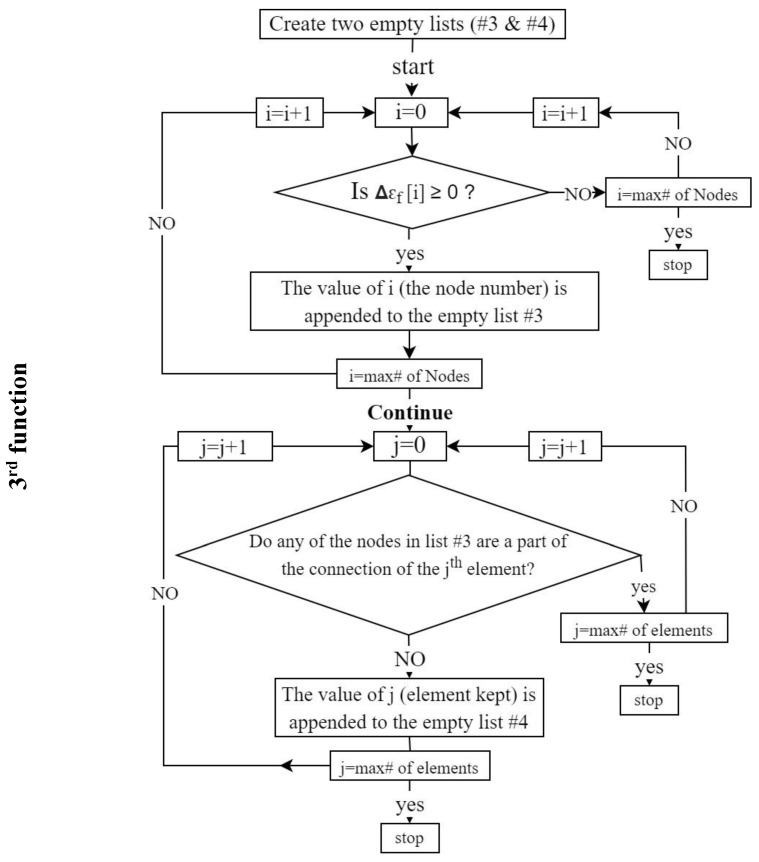
Flow chart describing the element deletion algorithm used to identify the designated numbers of nodes that should be deleted and the elements constituting the new model.

## Data Availability

The data presented in this study are available from the corresponding authors upon reasonable request.

## Appendix A

The framework of the code functions is depicted in detail in the following three flow charts:

The first flow chart describes the method in which the theoretical plastic strain at fracture of each node is calculated, the second one finds the difference between the FE and the theoretical plastic strain, and the last one identifies the designated numbers of nodes that should be deleted and the elements constituting the new model.
